# Validation of the vasoactive-ventilation-renal score in extreme preterm neonates

**DOI:** 10.1177/19345798251372550

**Published:** 2025-09-01

**Authors:** Eyad Bitar, Renjini Lalitha, Matthew Hicks, Aimann Surak, Abbas Hyderi, Dawn Pepper, Po Yin Cheung, Kumar Kumaran

**Affiliations:** 1 Division of Neonatology, Department of Pediatrics, 104820 Queen’s University, Kingston, ON, Canada; 2 Division of Neonatal-Perinatal Medicine, Department of Pediatrics, University of Western Ontario, London, ON, Canada; 3 Division of Neonatal-Perinatal Medicine, Department of Pediatrics, University of Alberta, Edmonton, AB, Canada

**Keywords:** ELGANs, morbidity, neonatology, outcomes, prematurity, VVR

## Abstract

**Objectives:**

To validate Vasoactive-Ventilation-Renal (VVR) score in extremely low gestational age neonates (ELGANs) as a predictor of mortality and morbidity by assessing its association with clinical outcomes.

**Study Design:**

This was a secondary analysis of data from a randomized controlled trial including neonates born 23^0^–28^6^ weeks’ gestation admitted to a Canadian tertiary-level neonatal intensive care unit between February 2019 and December 2021. VVR scores were measured at set intervals. Outcomes included mortality, intraventricular hemorrhage (IVH), bronchopulmonary dysplasia (BPD), necrotizing enterocolitis, patent ductus arteriosus, retinopathy of prematurity, mechanical ventilation duration, and length of hospital stay. Multivariate logistic regression analysis and receiver operating characteristic (ROC) curves were used to determine the association between VVR scores and clinical outcomes.

**Results:**

Data from 132 neonates were analyzed. The mean (SD) gestational age was 26.5 (1.5) weeks, and the mean (SD) birth weight was 933 (243) grams. A VVR score >48 was significantly associated with severe IVH (AOR: 5.8, 95% CI: 1.2–28.9, *p* = 0.03), BPD (AOR: 8.8, 95% CI: 1.1–72.4, *p* = 0.044), prolonged mechanical ventilation (>71 days) (AOR: 6.86, 95% CI: 1.6–30, *p* = 0.01), and extended hospital stay (>150 days) (AOR: 6.19, 95% CI: 1.4–26.4, *p* = 0.01). No significant associations were observed with mortality or other outcomes. ROC curves analysis demonstrated good predictive performance of VVR score at 7 days for these adverse outcomes.

**Conclusion:**

The VVR score at 7 days is a reliable predictor of significant adverse outcomes, including severe IVH and BPD, in ELGANs. Further studies in larger, diverse populations are warranted to confirm these findings.

## Introduction

The care of extremely low gestational age neonates (ELGAN) presents unique challenges and complexities for healthcare providers.^1^ These fragile neonates often require intensive medical intervention and close monitoring due to their immature organ systems.^2^ For optimal caregiving and resource allocation, predicting ELGAN’s clinical course and prognosis is crucial.^3^ Several prediction tools have been developed to forecast clinical outcomes accurately.^4–8^ These tools support healthcare providers in identifying risks, selecting the best course of action, and improving outcomes for neonates, particularly those with complex medical conditions. Common scoring systems include Apgar score, Clinical Risk Index for Babies (CRIB II), and Score for Neonatal Acute Physiology Perinatal Extension (SNAPPE II). These rely on static variables alongside biochemical parameters upon admission or a multitude of clinical variables shortly after birth.^9–13^ More recently, machine learning techniques that use complicated algorithms and models to analyze large datasets to predict clinical outcomes have been studied. None of these models exhibited a superior predictive capability compared to other studies, nor did they demonstrate notably high predictive effectiveness.^14,15^ The vasoactive-inotropic score (VIS) has been investigated and showed a correlation with outcomes after cardiac surgeries in infants and neonates.^16,17^ VIS reflects pharmacological support for the cardiovascular system and is determined as a weighted sum of administered inotropes and vasoconstrictors.^18^ However, ELGANs frequently have multi-organ dysfunction, affecting cardiovascular, respiratory and renal systems. A novel clinical tool, the Vasoactive-Ventilation-Renal (VVR) score, has come to light as a potential way to assess the severity of illness and predict clinical outcomes.^19^ It incorporates markers of cardiovascular, pulmonary, and renal function, perhaps the three organ systems most frequently impacted in ELGAN. VVR is calculated as follows: VIS + ventilation index (VI) + (change in serum creatinine (Cr) from baseline X 10). In the pediatric populations, the VVR score strongly correlated with the duration of mechanical ventilation, length of hospital stay and mortality post-cardiac surgery.^20,21^ In neonates who underwent cardiac surgery, the VVR score outperformed conventional severity scores.^22,23^ To date, there has been no study aiming to validate this instrument in the context of premature neonates for the prediction of morbidity and mortality. This study aims to validate the VVR score in a cohort of ELGAN to evaluate its accuracy and reliability as a prognostic tool.

## Objectives

To validate the VVR score as a predictor of clinical outcomes in ELGAN by investigating the association between VVR score and mortality and morbidity.

## Methods

### Study design

This study was a secondary analysis of data collected from a single-center randomized controlled trial (RCT) (ClinicalTrials.gov Identifier - NCT03841929) that examined the effect of multimodal monitoring of hemodynamics in ELGAN in a Canadian tertiary-level unit.^24^ The aim of that RCT was to investigate whether a multimodal assessment consisting of targeted neonatal echocardiogram, cerebral near-infrared spectroscopy (NIRS) and clinical-biochemical data could reduce VVR scores in ELGAN within the first week of life.

### Participants and procedures

In the main study, participants were premature neonates born between 23° and 28^6^ gestational age (GA) and admitted between February 2019 and December 2021 to the Stollery Philip C. Etches Neonatal Intensive Care Unit (NICU) at Royal Alexandra Hospital in Edmonton, Canada, a 69-bed unit specializing in the care of ELGANs, with access to a neonatal hemodynamic consultation service. Exclusion criteria included neonates with structural heart disease and major congenital or chromosomal disorders. After applying inclusion and exclusion criteria, participants were randomized to receive comprehensive hemodynamic monitoring that included continuous cerebral Near Infrared Spectroscopy (NIRS) monitoring for the first 72 h of life, Targeted Neonatal Echocardiography (TNE) performed at 18–24 h and 66–72 h of life and a hemodynamic consultation service, or a control arm that involved the standard of care practice. A simple computer-based randomization and allocation concealment methods were used in this study. Participants were followed until they were discharged home. The study protocol, intervention, informed consent, and related materials were approved by the Research Ethics Board at the University of Alberta (Pro00084238).

### Measures

VVR scores were recorded at randomization and then every 6 h for the first 72 h of life. The score was also measured at 7 days of age. The following patient demographics were acquired through patient chart review: GA, birth weight (BW), sex, type of pregnancy, mode of delivery, antenatal steroids, prolonged rupture of membrane, suspected or confirmed chorioamnionitis, delayed cord clamping, and Apgar scores. The clinical outcomes of interest were death, intraventricular hemorrhage (IVH), severe IVH (defined as grade 3 or 4 IVH (IVH with ventricular enlargement and/or parenchymal echogenicity)), pulmonary hemorrhage (PH), bronchopulmonary dysplasia (BPD) defined as oxygen or respiratory support at 36 weeks corrected gestational age, necrotizing enterocolitis (NEC) Bell stage 2 or 3, retinopathy of prematurity (ROP), patent ductus arteriosus (PDA), length of hospital stay and duration of mechanical ventilation.

### Calculation of VVR score

The VVR score was calculated using the following formula: VVR = VIS + VI + (Δ Cr x 10).

VIS was calculated based on the doses of vasoactive medications administered, as follows: VIS = dopamine (mcg/kg/min) + dobutamine (mcg/kg/min) + (100 × epinephrine (mcg/kg/min)) + (10 × milrinone (mcg/kg/min)) + (10,000 × vasopressin (unit/kg/min)) + (100 × norepinephrine (mcg/kg/min)). VIS was recorded as zero for patients receiving no vasoactive support at the time of VVR measurement.

VI was calculated to quantify ventilatory support requirements using the formula: VI = (Ventilator RR × (PIP−PEEP) × PaCO2)/1000.

RR: respiratory rate, PIP: peak inspiratory pressure, PEEP: positive end-expiratory pressure. PaCO2: partial pressure of carbon dioxide.

For patients on Non-Invasive Mechanical Ventilation (NIMV), a modified PIP value was used, calculated by reducing the set PIP by 5 cm H_2_O to account for pressure attenuation reaching the nares. For patients on nasal CPAP (continuous positive airway pressure), the VI was recorded as zero. For those on High-Frequency Ventilation, the difference between PIP and PEEP was replaced by Mean Airway Pressure, with a standard ventilator rate of 60 breaths per minute (maximum rate on conventional mechanical ventilation) applied for calculation purposes. Where arterial blood gas values for PaCO_2_ were unavailable, transcutaneous CO_2_ measurements were adjusted by subtracting 5 mmHg to approximate arterial CO_2_ levels.

ΔCr represented the change in serum creatinine from baseline values within the first 7 days of life. To calculate ΔCr, baseline serum creatinine was subtracted from subsequent creatinine values within the defined time frame. If repeat creatinine measurements were less than or equal to baseline levels, ΔCr was recorded as zero. Serum creatinine was measured in mg/dL. For values initially measured in mmol/L, conversion to mg/dL was performed using the formula: serum creatinine (mg/dL) = serum creatinine (mmol/L) × 0.0113.

### Statistical analysis

Descriptive statistics, such as means, standard deviations, medians, quartiles, numbers, and proportions were calculated where relevant. The study employed logistic regression to examine the association between VVR scores and important outcomes, conducting both unadjusted and adjusted analyses for the intervention arm in the main RCT. The odds ratio was used as the measure of association, indicating the likelihood of outcomes in relation to VVR scores. In order to classify the infants into groups with high and low risk, a VVR cutoff was employed for the analyses. This cutoff was chosen pragmatically based on the 95th percentile in this study’s population to identify infants with the greatest physiological instability. While this method aligns with previous studies using local data when external references are lacking,^25^ the authors acknowledge that such a cutoff may limit generalizability. The proportions of infants with VVR scores above the predefined threshold of 48 (95^th^ centile) were measured, and the significance of these proportions was tested using the Chi-square test. Thresholds for prolonged mechanical ventilation and extended hospital stays were based on the 75th percentile of these variables within our study cohort. This is to identify infants in the highest quartile of resource utilization and illness severity. This approach aligns with prior studies that have operationalized prolonged support by defining thresholds relative to the study population distribution when external standard cutoffs are lacking.^26,27^ A p-value of 0.05 was used as the threshold for statistical significance. Finally, to present the predictive capacity of VVR scores, the Receiver Operating Characteristic (ROC) curves for VVR scores in relation to important clinical outcomes were illustrated.

The statistical analyses were conducted using the STATA statistical software (Stata 18.0). All data were securely stored on an institutional computer protected by password access. The database was constructed using university-approved REDCap software.

## Results

**Figure 1.**
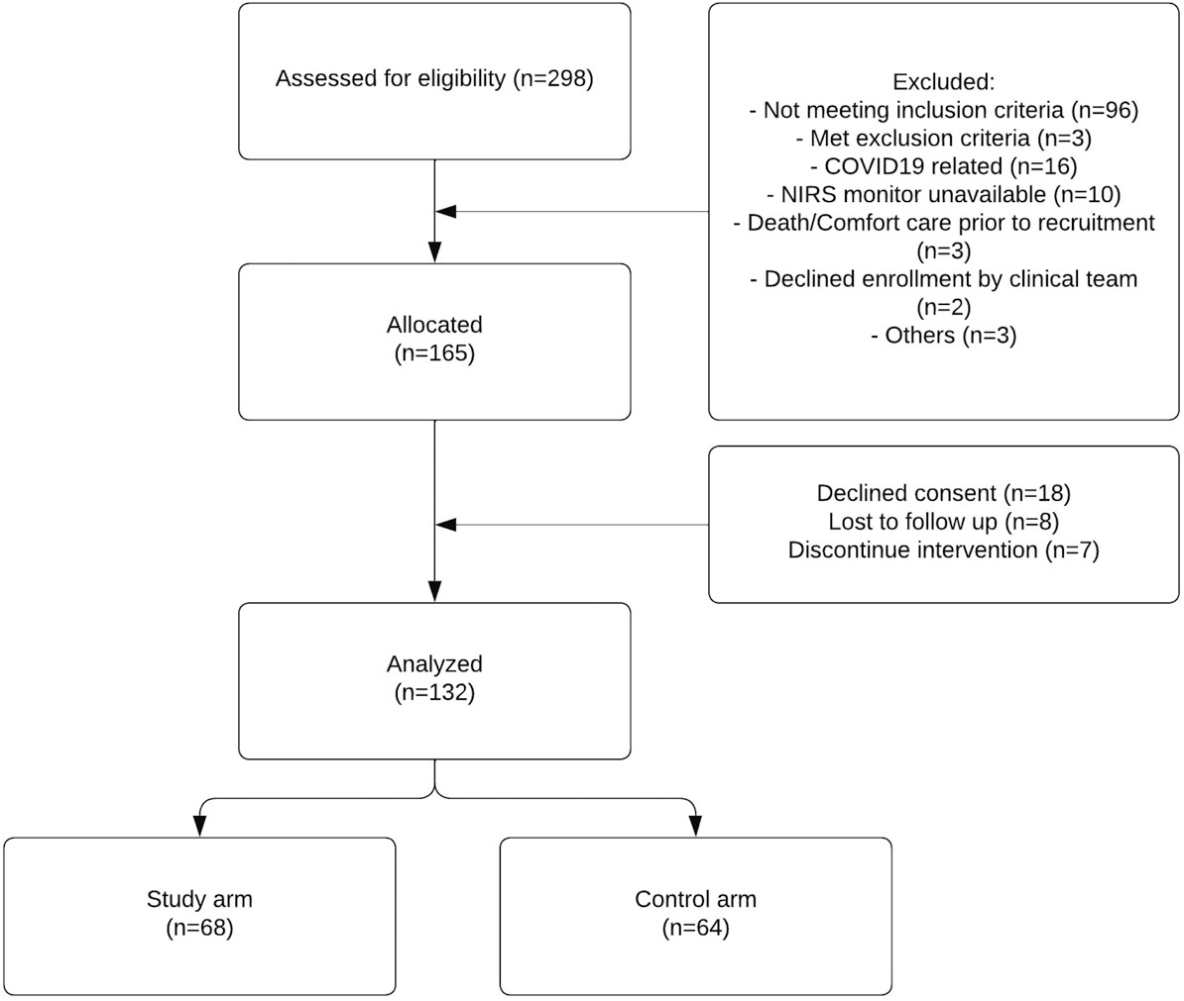
Enrollment flow chart.

**Figure 2.**
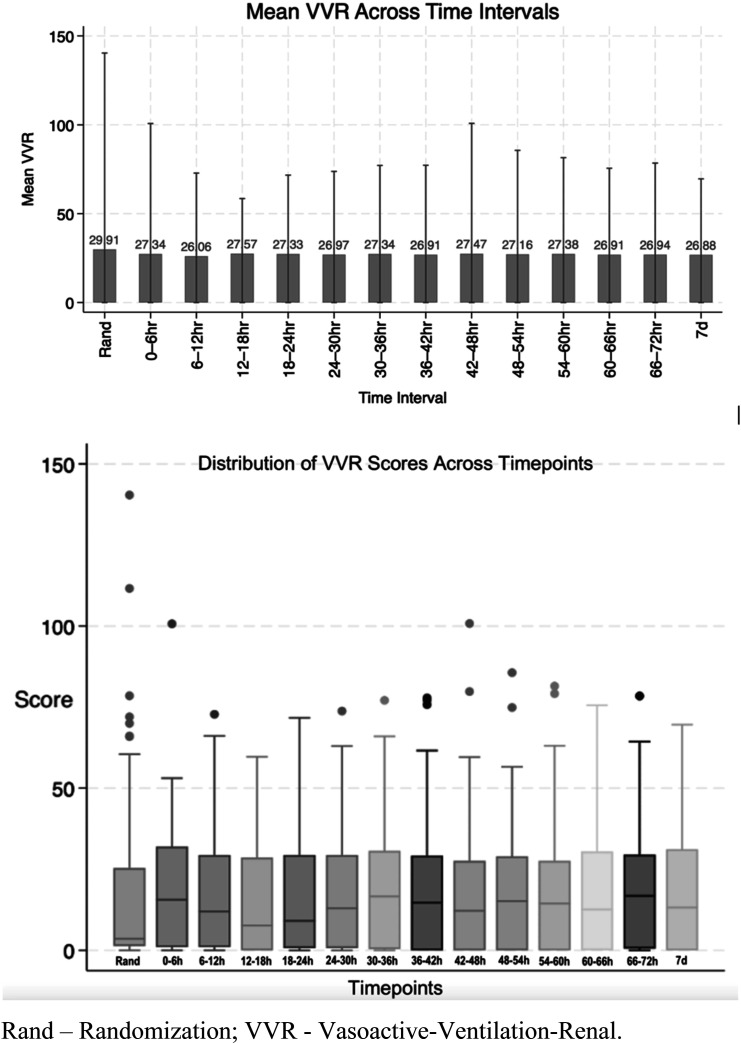
Vasoactive-ventilation-renal (VVR) scores over time.

**Figure 3.**
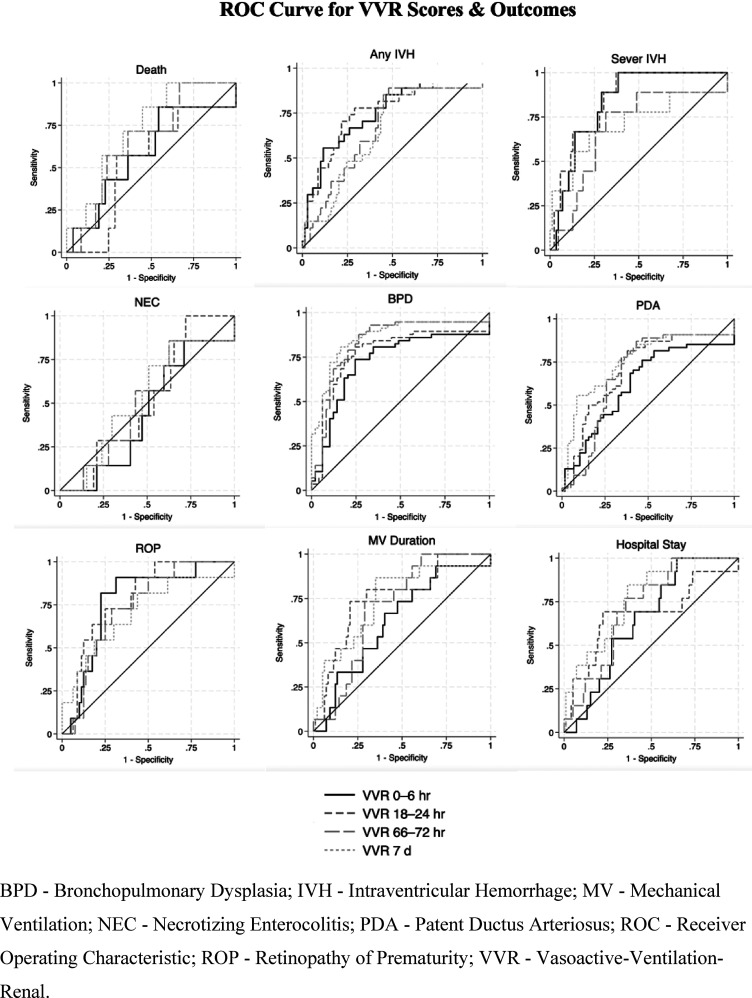
Receiver Operating Characteristic (ROC) curves for VVR scores & outcomes.

Of 298 neonates screened for eligibility, 165 neonates were allocated to study arms. Of those, 132 neonates were analyzed (68 in the study arm and 64 in the control arm). Due to the deferred consent nature, data were not available for neonates where consent was declined or lost to follow-up after allocation (Figure 1). Among the neonates included in the analysis, the mean GA (SD) was 26.5 (1.5) weeks, and the average BW (SD) was 933 (243) grams. Table 1 provides an overview of the key demographic and clinical characteristics of the individuals who participated in the study. Mean VVR scores ranged between 15 and 18.3 in the first 7 days of life. Figure 2 summarizes the measured VVR scores between randomization and 7 days of age. Table 2 presents the neonatal outcomes observed in the study cohort. The mean VVR score at 7 days was 17.7 (SD 17.9), with a median score of 13.2 (IQR 0–31.2). Additionally, 8.5% of neonates had a VVR score >48 at 7 days, and inotropic support was utilized in 19.7% of cases. Death occurred in 6.8% of the study population between 7 and 44 days of age. Table 3 displays the odds ratios for various neonatal outcomes associated with an elevated VVR score (>48) at 7 days. Adjusted and unadjusted odds ratios are presented at different time points (0–6 h, 24 h, 72 h, and 7 days). The results indicate an association between elevated VVR score (>48) at 7 days and the following outcomes: severe IVH (AOR: 5.8, 95% CI: 1.2–28.9, *p* = 0.03), BPD (AOR: 8.8, 95% CI: 1.1–72.4, *p* = 0.044), mechanical ventilation (MV) duration >71 days (AOR: 6.86, 95% CI: 1.6–30, *p* = 0.01) and extended hospital stay >150 days (AOR: 6.19, 95% CI: 1.4–26.4, *p* = 0.01). Furthermore, Table 4 examines the association between an elevated VVR score (>48) at 7 days and neonatal outcomes. A statistically significant association was observed for the following outcomes: severe IVH (grade 3–4) (*p* = 0.01), BPD (*p* = 0.008), PDA treatment (*p* = 0.04), hospital stay (*p* = 0.003) and MV duration (*p* = 0.003). Table 5 presents the AUC values of the VVR score at different time points (0–6 hours, 24 hours, 72 hours, and 7 days) for predicting major neonatal outcomes. Figure 3 displays the ROC curves, evaluating the effectiveness of VVR scores in predicting important clinical outcomes. ROC curves demonstrated that VVR scores at 7 days of age were more effective in predicting clinical outcomes than earlier scores. Table 6 presents diagnostic accuracy metrics of the VVR score at 7 days, including AUC with 95% CI, sensitivity, specificity, and likelihood ratios, for predicting key neonatal outcomes.

**Table 1.** Baseline characteristics of study participants.

Characteristics	
Gestational age in weeks, (SD)	26.5 (1.5)
Birth weight, grams (SD)	933 (243)
Male sex, n (%)	75 (56.8)
Vaginal delivery n (%)	46 (34.9)
Antenatal steroids, n (%)	124 (94.7)
PROM >18 h, n (%)	48 (36.4)
Suspected/confirmed chorioamnionitis, n (%)	14 (10.8)
Apgar score@5 min less than 7, n (%)	55 (41.7)
DCC >30 s, n (%)	75 (69.4)
SNAPEII score, (SD)	32.2 (19.5)

DCC - Delayed Cord Clamping; PROM - Prolonged Rupture of Membrane; SD - Standard Deviation; SNAPPEII - Score for Neonatal Acute Physiology Perinatal Extension-II.

**Table 2.** Neonatal outcomes.

Outcome	
Death, n (%)	9 (6.8)
IVH any grade, n (%)	29 (25.2)
Severe IVH (grade 3–4), n (%)	10 (8.7)
BPD, n (%)	62 (50.0)
NEC, n (%)	8 (6.1)
ROP laser surgery, n (%)	11 (10.2)
PDA treated, n (%)	58 (43.9)
Length of hospital stay in days, (SD)	103.0 (90.2)
Days of mechanical ventilation, (SD)	33.7 (84.8)
Mean VVR score at 7 days (SD)	17.7 (17.9)
Median VVR score at 7 days (IQR)	13.2 (0–31.2)
VVR score at 7 days >48, n (%)	11 (8.5)
Change in VVR over 7 days, SD	1.2 (25.5)
Use of inotropes in first 7 days, n (%)	26 (19.7)

BPD - Bronchopulmonary Dysplasia; IVH - Intraventricular Hemorrhage; IQR - Interquartile Range; NEC - Necrotizing Enterocolitis; PDA - Patent Ductus Arteriosus; ROP - Retinopathy of Prematurity; SD - Standard Deviation; VVR - Vasoactive-Ventilation-Renal.

**Table 3.** Odds ratio for the outcome for VVR >48.

Variable	VVR score 0–6 h				VVR score 24 h				VVR score 72 h				VVR score 7 days			
	Unadjusted		Adjusted		Unadjusted		Unadjusted		Unadjusted		Adjusted		Unadjusted		Adjusted	
	OR	p-value	OR	p-value	OR	p-value	OR	p-value	OR	p-value	OR	p-value	OR	95%CI	OR	95%CI
Death	2.9	0.22	2.7	0.25	1	-	1	-	0.97	0.98	1.01	0.99	1.4	0.2–12.5	1.9	0.2–18.6
IVH any	1.1	0.87	1.2	0.84	2.03	0.25	2.44	0.17	0.71	0.62	0.7	0.61	2.1	0.6–8.2	1.9	0.5–7.6
IVH severe	1.04	0.97	1.07	0.95	4.03	0.07	5.26	0.045	0.71	0.76	0.71	0.75	5.9	1.3–28.1	5.8	1.2–28.9
BPD	0.8	0.75	0.84	0.78	0.84	0.77	1.04	0.95	0.52	0.26	0.47	0.21	10.4	1.3–84.5	8	1.1–72.4
NEC	1	-	1	-	1	-	1	-	1.11	0.92	1.11	0.92	1	-	1	-
ROP Tx	0.87	0.9	0.94	0.95	1	-	1	-	1	-	1	-	2.9	0.5–15.9	2.2	0.5–15.9
PDA Tx	1.56	0.45	1.63	0.41	0.35	0.12	0.38	0.16	0.17	0.022	0.16	0.02	3.8	1–15	3.5	0.9–14.1
Hospital stays (>150 days)	1	-	1	-	0.68	0.72	0.75	0.8	1.35	0.72	1.32	0.74	6.34	1.6–25.4	6.19	1.4–26.4
MV duration (>71 days)	1	-	1	-	0.63	0.66	0.6	0.64	0.53	0.55	0.53	0.55	5.7	1.4–22.6	6.86	1.6–30

BPD - Bronchopulmonary Dysplasia; IVH - Intraventricular Hemorrhage; MV - Mechanical Ventilation; NEC - Necrotizing Enterocolitis; OR - Odds Ratio; PDA Tx - Patent Ductus Arteriosus Treatment; ROP Tx - Retinopathy of Prematurity Treatment; SD - Standard Deviation.

**Table 4.** Elevated VVR >48 at 7 days and neonatal outcomes.

Variable	Total	VVR >48, *N* (%)	VVR <48, *N* (%)	*p*-value
Death, n (%)	9 (6.2%)	1 (9%)	8 (6.6%)	0.75
IVH any grade, n (%)	29 (25.2%)	4 (40%)	25 (23.8%)	0.26
Severe IVH (grade 3–4), n (%)	10 (8.8%)	3 (30%)	7 (6.7%)	0.01
BPD, n (%)	62 (50%)	9 (90%)	53 (46.5%)	0.008
NEC, n (%)	8 (6.1%)	0 (0%)	8 (6.7%)	0.38
ROP Tx, n (%)	11 (10.2%)	2 (22.2%)	9 (9%)	0.75
PDA Tx, n (%)	58 (43.9%)	8 (72.7%)	50 (41.3%)	0.04
Hospital stays, mean (SD)	131	180.27 (90.2)	59.9 (81.9)	0.003
MV duration, mean (SD)	130	105.8 (142.9)	27 (74.8)	0.003

BPD - Bronchopulmonary Dysplasia; IVH - Intraventricular Hemorrhage; MV - Mechanical Ventilation; NEC - Necrotizing Enterocolitis; PDA Tx - Patent Ductus Arteriosus Treatment; ROP Tx- Retinopathy of Prematurity Treatment; SD - Standard Deviation; VVR - Vasoactive-Ventilation-Renal.

**Table 5.** Area Under the Curve (AUC) for VVR Scores at different time points predicting neonatal outcomes.

Outcome	AUC			
	0–6 h VVR	18–24 h VVR	66–72 h VVR	7 days VVR
Death	0.59	0.56	0.59	0.7
IVH (any grade)	0.76	0.77	0.69	0.68
Severe IVH	0.82	0.87	0.72	0.76
BPD	0.73	0.80	0.85	0.87
NEC	0.46	0.54	0.52	0.55
ROP Tx	0.76	0.80	0.72	0.72
PDA Tx	0.64	0.72	0.69	0.79
Hospital stays (>150 days)	0.61	0.67	0.72	0.73
MV duration (>71 days)	0.6330	0.7654	0.6969	0.7588

BPD - Bronchopulmonary Dysplasia; IVH - Intraventricular Hemorrhage; MV - Mechanical Ventilation; NEC - Necrotizing Enterocolitis; PDA Tx - Patent Ductus Arteriosus Treatment; ROP Tx- Retinopathy of Prematurity Treatment; SD - Standard Deviation; VVR - Vasoactive-Ventilation-Renal.

**Table 6.** Diagnostic accuracy metrics of VVR Score at 7 Days for predicting neonatal outcomes.

Outcome	AUC (95%CI)	Sensitivity	Specificity	LR+	LR−
Death	0.7 (0.56–0.83)	0.11	0.93	1.49	0.96
IVH (any grade)	0.68 (0.58–0.79)	0.14	0.95	2.82	0.91
Severe IVH	0.76 (0.57–0.95)	0.30	0.92	3.61	0.76
BPD	0.87 (0.81–0.94)	0.15	0.88	1.25	0.97
NEC	0.55 (0.38–0.72)	—	1.00	—	1.00
ROP Tx	0.72 (0.56–0.89)	0.18	0.91	1.98	0.90
PDA Tx	0.79 (0.71–0.87)	0.14	0.97	5.11	0.89
Hospital stays (>150 days)	0.73 (0.59–0.88)	0.36	0.96	8.50	0.67
MV duration (>71 days)	0.76 (0.63–0.89)	0.33	0.96	7.74	0.70

BPD - Bronchopulmonary Dysplasia; IVH - Intraventricular Hemorrhage; MV - Mechanical Ventilation; NEC - Necrotizing Enterocolitis; PDA Tx - Patent Ductus Arteriosus Treatment; ROP Tx- Retinopathy of Prematurity Treatment; SD - Standard Deviation; VVR - Vasoactive-Ventilation-Renal.

## Discussion

This analysis of ELGAN provides valuable insights into the potential of the VVR score as a prognostic tool. To our knowledge, this is the first study to evaluate and validate the VVR score in extremely low gestational age neonates, extending its application beyond post-cardiac surgery populations. In this cohort, the VVR score exhibited significant associations with specific morbidities that are critical in the care of ELGANs. These findings suggest that VVR measurement at 7 days could be incorporated into ongoing risk stratification, allowing clinicians to identify neonates who may benefit from closer monitoring and targeted interventions. Notably, neonates with elevated VVR scores at 7 days of age were more likely to experience severe IVH. Elevated VVR scores may reflect greater physiologic instability and multi-organ stress in ELGANs, which may be associated with an increased risk for adverse outcomes such as IVH.^28^ Additionally, respiratory insufficiency, often indicated by increased ventilation requirements captured in the VVR score, may exacerbate cerebral hypoxia and ischemia, further compromising cerebral autoregulation and increasing the risk of IVH.^29^ However, without fraction of inspired oxygen (FiO_2_) or oxygen saturation (SpO_2_) data, a direct link to cerebral hypoxia or ischemia cannot be inferred. In addition, most cases of severe IVH in ELGANs occur within the first week of life; therefore, the predictive value of an elevated score measured at 7 days may reflect ongoing hemodynamic instability or evolving multi-organ dysfunction in infants who have already developed or are at imminent risk of IVH. Clinically, this association is still relevant: a high score at 7 days may help identify infants who remain physiologically vulnerable and who might benefit from closer neurological surveillance. Furthermore, elevated VVR scores at 7 days were associated with a higher incidence of BPD. This may be because neonates with elevated VVR scores often have more pronounced cardiovascular compromise or respiratory insufficiency, predisposing them to lung injury.^30^ Additionally, the use of respiratory support, particularly mechanical ventilation, may further contribute to lung injury and inflammation, exacerbating BPD risk in susceptible neonates. In addition, this association may reflect underlying systemic inflammation or oxidative stress, both of which play key roles in the pathogenesis of BPD. These processes may also be reflected in elevated VVR scores.^31^ This association alone does not confirm the validity of the VI component as a predictive marker. Instead the VI may serve as a surrogate indicator of overall respiratory burden and severity of illness in ELGANs, and its predictive role for BPD warrants further validation. These findings are in congruence with previous research linking illness severity scores to adverse outcomes in extremely premature neonates. Similarly, SNAP-II showed significant and independent predictability for severe IVH and BPD.^32^ In addition, our study observed an association between VVR scores at 7 days and the need for a longer duration of mechanical ventilation and prolonged length of hospital stay. This may reflect the hemodynamic instability and compromised respiratory and cardiovascular function in some ELGANs that may necessitate more intensive medical management and supportive care. This correlates with previous findings suggesting a link between VIS and the duration of mechanical ventilation and length of hospital stay in neonates after cardiac surgery.^20,21^

Interestingly, VVR scores measured at earlier time points did not exhibit as robust an association with outcomes when compared to those measured at 7 days. This raises compelling questions about the underlying mechanisms driving this occurrence. One possible explanation is that VVR scores at 7 days capture a more comprehensive picture of the neonate’s physiological status and response to postnatal challenges. In the early neonatal period, infants may still be undergoing significant physiological adaptation, and their clinical trajectory may not yet be fully established. As a result, VVR scores measured at earlier time points may not accurately reflect the neonate’s long-term prognosis or susceptibility to adverse outcomes. By contrast, VVR scores measured at 7 days likely encompass a critical period during which the neonate’s physiological stability and response to therapeutic interventions become more evident. However, the fact that VVR score was predictive at 7 days but not in the first 72 h limits its use for immediate postnatal risk assessment, making it better suited for ongoing rather than initial prognostication.

While existing prognostic tools like CRIB II and SNAP-II are valuable for predicting outcomes in ELGANs, the VVR score offers unique contributions and potential advantages. Unlike CRIB II, which primarily focuses on assessing mortality risk based on BW, GA and clinical status at NICU admission, and SNAP-II, which evaluates acute physiological status, the VVR score integrates markers of cardiovascular, pulmonary, and renal function. By encompassing these critical organ systems, the VVR score provides a more comprehensive assessment of the neonate’s physiological status, offering insights into the complex interplay between hemodynamics, ventilation, and renal function. Additionally, the VVR score’s incorporation of serial measurements over time allows for dynamic monitoring of clinical progression, potentially capturing subtle changes in the neonate’s condition that may not be evident with single-time-point assessments.

Incorporating the VVR score into clinical practice offers valuable insights into the prognosis of ELGANs, but it comes with practical challenges. Variations in available resources, like monitoring equipment, and differences in electronic health record systems could affect implementation across different healthcare settings. Healthcare providers would need training to accurately calculate and interpret the VVR score. Standardizing data collection methods across institutions is essential for consistent results. Despite these challenges, the potential benefits of the VVR score in guiding clinical decisions for ELGANs highlight the importance of addressing these obstacles through further research and collaboration.

Including VIS in the VVR score may be less useful for ELGANs, who receive inotropes less often than post-cardiac surgery infants for whom VIS was originally designed.^33^ Also, excluding hydrocortisone, a common vasoactive drug in ELGANs, may underestimate cardiovascular support and reduce accuracy. Future research should consider adapting VIS to include such agents for better prediction in this population. Additionally, the method used to calculate VI may underestimate the respiratory burden in HFOV compared to conventional ventilation and does not account for FiO_2_, an important indicator of illness severity. Refining the VI calculation to incorporate FiO_2_ could enhance its accuracy and clinical relevance in ELGANs. Finally, the ΔCr component may be less reliable in ELGANs because early creatinine levels often reflect maternal rather than neonatal kidney function,^34^ limiting its value for early risk prediction. Alternative renal markers or adjusting baselines could improve the precision of the score.

Several limitations of this study should be acknowledged. The relatively small sample size may have limited the statistical power to detect differences in other outcomes, such as mortality. Additionally, this study was a secondary analysis of data from a single-institution randomized controlled trial with specific inclusion and exclusion criteria, potentially limiting its generalizability to a broader population of ELGANs. Being part of an interventional trial may have influenced clinical care and VVR scores. Excluding infants with structural heart diseases and major congenital or chromosomal disorders from the original RCT may have led to a limited representation of the ELGAN population, potentially overlooking subsets of infants at higher risk for adverse outcomes. To confirm the VVR score’s broader applicability, multicenter studies including more diverse populations are needed.

## Conclusion

In conclusion, while the VVR score did not independently predict overall mortality in this study, the score at 7 days was a promising predictor of adverse outcomes, such as severe IVH and BPD. This association with specific morbidities suggests its potential as a complementary tool in risk stratification and early intervention for ELGANs. Our findings indicate that integrating the VVR score into clinical practice could help identify neonates at higher risk of adverse outcomes, enabling targeted interventions to mitigate these risks and improve long-term outcomes. However, further research is necessary to validate these findings in larger cohorts of ELGANs, considering the limitations of our study. Ultimately, the VVR score holds promise as part of a comprehensive approach to ELGAN care, facilitating tailored interventions to enhance long-term outcomes and the quality of life for these vulnerable neonates.

## Appendix

### Abbreviations

BPD: Bronchopulmonary Dysplasia
BW: Birth Weight
ELGANs: Extremely Low Gestational Age Neonates
GA: Gestational Age
IVH: Intraventricular Hemorrhage
NEC: Necrotizing Enterocolitis
NICU: Neonatal Intensive Care Unit
PDA: Patent Ductus Arteriosus
RCT: Randomized Controlled Trial
ROP: Retinopathy of Prematurity
VIS: Vasoactive-Inotropic Score
VVR: Vasoactive-Ventilation-Renal
